# Mild Influenza in Children

**Published:** 1909-08-28

**Authors:** John Allan


### The General Practitioner's Column.

[Contributions to this Column are invited, and if accepted will be paid for.]

### MILD INFLUENZA IN CHILDREN.

By JOHN ALLAN, M.D.(Edin.).

In the course of general practice many apparently trifling ailments in children are met with, and these ailments may be puzzling both as regards diagnosis and treatment. In not a few cases the illness appears so slight that medical aid is not sought at the time, and it is for the after-effects that advice is asked. I believe that many of these children suffer from mild influenza (which is, perhaps, not recognised as such), an opinion borne out by the condition of lassitude which persists for some time.

The treatment will differ according as one sees the case during or after the attack. If the child is seen when the attack is in progress the history is probably as follows. The child has been listless and feels tired. On examination there is little to be made out. The lassitude is obvious. The temperature, if it is raised at all, is only slightly so, and rarely exceeds 100° F. This is somewhat surprising, because the temperature in children often rises to 102° or 103° F. on very slight provocation.

With regard to treatment the first point is that the child should be kept in bed for a day or two. The irregularity of the heart, which, if carefully looked for, will be found in practically all cases, indicates that there is some underlying condition which should not be lightly treated. With prompt and efficient rest it is marvellous how quickly the heart recovers in these cases. After about forty-eight hours the child can be allowed up and permitted to move about the house, and in a few days' time he is well enough to play and romp about. In these mild cases treated by rest cardiac stimulants are not necessary, and during convalescence a general tonic containing iron and arsenic will act effectively.

The diet for the first day or two will of necessity be light, because the child is disinclined for food and may be sick. Therefore, milk, beef-tea, custard, chicken jelly, and the like should be given. As soon, however, as the stomach can tolerate more solid food, the diet should be increased, and the child should be encouraged to eat as much as possible. A generous, nutritious, and easily digested diet will do more to hasten convalescence than almost anything else.

Many drugs have been advised. The late Sir William Broadbent believed in quinine. His favourite prescription was ammoniated tincture of quinine and liquor ammoniæ acetatis. In the presence of an epidemic of influenza he had great faith in two-grain doses of quinine once daily as a prophylactic measure. Sodium salicylate is preferred by some. Dr. T. W. Banks, a practitioner in Lanarkshire, treats his cases of influenza by giving the patients powders of salicylate of soda and antefebrin. His experience is that three powders, one every four hours, will check the attack. In my opinion both quinine and salicylate of soda are objectionable for children, because the intensely bitter taste of quinine and the sweet mawkish taste of salicylate of soda are not such as to commend these drugs to them. It is advisable to prescribe for children drugs which have not pronounced tastes. In acetyl-salicylic acid we have a drug which is easily taken and is efficacious. The drug may either be given in powder or in a mixture flavoured with orange or lemon syrup. The latter method is to be preferred for children. An initial dose of 15 grains should be given when the child goes to bed; then 10 grains every six hours for the next 24 hours, and thereafter for the next few days, 5 grains thrice daily. This dosage is suitable for a child ten years of age. It may be mentioned that a child need not be kept in bed because he is taking 5 grains of acetyl-salicylic acid three times a day, as in these doses no sweating or discomfort is caused. As a substitute for quinine euquinine appears to be excellent, and those who have had much experience with it speak highly of it. I myself have only used it in a few cases, but always with satisfactory results. In one case of mild influenza in a child 5 grains of euquinine four times a day were given, and appeared to act efficiently. The drug, therefore, would appear worthy of a trial. It is stated to be incompatible with acids.

Let us now consider the treatment of mild influenza when advice is sought a week or two after the actual attack. The history is that ten days before the child felt tired and complained of a feeling of general soreness throughout the body. There was anorexia, and the child perhaps vomited on one or two occasions.

On examination absence of alertness and lack of energy are marked features, and on being asked to walk once or twice round the room the child will soon begin to drag his legs. The child appears in general terms to be suffering from debility. On auscultation cardiac irregularity is quite apparent, and in some cases bruits can be detected.

The child should be put to bed for a few days. There is at this stage no necessity to limit the diet to fluids, and every effort should be made to encourage the child to eat. Moderate doses of acetyl-salicylic acid are distinctly beneficial and should certainly be prescribed for a few days. The heart will not now quickly respond to rest simply, and it is advisable to give small doses of strophanthus and strychnine to stimulate the cardiac muscle. Four minims of tincture of strophanthus (B.P., 1885) and one or two minims of liquor strychninæ will generally suffice for a child of ten. During convalescence tonics should be given, and the child should not be forced to indulge in undue exertions for some weeks.

Influenza seems to have a special affinity for the heart in the child, and the severity of the attack is no criterion of the damage that may be done to that organ. Indeed, my experience has been that the mild evanescent attacks of influenza are just as likely to affect the heart as the severe ones. With careful treatment there is no reason to fear permanent damage to the heart, but neglect is only too likely to result in irretrievable injury and to cripple the child when he reaches adolescence and manhood.

